# Late-Onset Digital Ischaemia after WALANT: Successful Reversal with Off-Label Urapidil

**DOI:** 10.1055/a-2739-3810

**Published:** 2025-12-11

**Authors:** Andrea Frey, Lukas Mathys, Christine Kammerhofer

**Affiliations:** 1 Hand Surgery, Kantonsspital Basel, Basel, Switzerland

**Keywords:** WALANT, digital ischaemia, urapidil, epinephrine, hand surgery

## Abstract

**Background:**

WALANT (Wide Awake Local Anaesthesia No Tourniquet) has revolutionised hand surgery by enabling procedures with a combination of lidocaine and epinephrine, thereby eliminating the need for sedation or a tourniquet. Although epinephrine is now considered safe in low concentrations, rare ischaemic complications may still occur.

**Case Report:**

This report concerns a 74-year-old woman who developed delayed-onset digital ischaemia after WALANT-assisted excision of a Dupuytren’s nodule. Phentolamine, the standard reversal agent, was unavailable. Local infiltration with urapidil (12.5 mg), a selective α₁-adrenergic antagonist, rapidly restored perfusion and sensation. The patient recovered fully without sequelae.

**Conclusion:**

This case highlights a rare but critical complication of WALANT. Urapidil may represent a safe and effective alternative to phentolamine in the management of epinephrine-induced vasospasm.

## Introduction

The WALANT (Wide Awake Local Anesthesia No Tourniquet) technique, first described by Lalonde 1 , has fundamentally changed modern hand surgery practice. By combining lidocaine with low-dose epinephrine (typically 1:100,000), surgeons achieve both effective anesthesia and hemostasis without the need for sedation or tourniquet use. The technique allows patients to remain awake and cooperative, enabling real-time intraoperative assessment of active motion, which is particularly useful for tendon and nerve procedures. WALANT has been increasingly adopted worldwide due to its cost-effectiveness, reduced perioperative morbidity, and the ability to perform procedures in minor procedure rooms outside traditional operating theaters 2 , 3 .

Epinephrine serves a dual role within the WALANT protocol. Its vasoconstrictive effect significantly reduces intraoperative bleeding, providing a clear surgical field and potentially shortening operative time. Moreover, by decreasing local blood flow, epinephrine slows systemic absorption of lidocaine, thereby prolonging its anesthetic effect and enhancing patient comfort throughout the procedure. The anesthetic duration typically ranges from 4 to 6 hours, depending on tissue characteristics and injection technique. The maximal vasoconstrictive effect generally occurs 20 to 30 minutes after infiltration, underscoring the importance of adequate waiting time before incision to achieve optimal hemostasis 3 .

Historically, concerns existed regarding the risk of digital necrosis when epinephrine was used in the fingers or toes, largely due to reports involving high-concentration solutions or unbuffered preparations 4 , 5 . However, numerous studies in the last two decades have confirmed the safety of appropriately buffered, low-concentration epinephrine in digital blocks, demonstrating extremely low rates of ischemic complications (<0.1%) even in elderly patients or those with mild vascular comorbidities 3 , 6 .

Despite its overall safety, WALANT requires careful patient selection. Contraindications include known peripheral vascular disease, Raynaud’s phenomenon, Buerger’s disease, prior digital replantation, severe arteriosclerosis, uncontrolled hypertension, and systemic conditions that may compromise microvascular circulation 5 , 7 . Age-related endothelial dysfunction and reduced microvascular reserve may increase susceptibility to vasospastic events even in patients without overt vascular disease. Preoperative assessment should include a detailed vascular history and consideration of alternative anesthetic strategies in high-risk patients.

While phentolamine, a non-selective α-adrenergic antagonist, remains the standard reversal agent for epinephrine-induced digital ischemia, access may be limited in some institutions. Alternative pharmacologic approaches, including selective α₁-blockers such as urapidil, are worth considering. Understanding the mechanisms of vasospasm, the pharmacology of reversal agents, and the risk factors for ischemia is critical to ensuring patient safety during WALANT procedures 7 , 8 .

## Case Report

A 74-year-old right-handed woman presented with a Dupuytren’s nodule at the base of her right middle finger. Past medical history included well-controlled hypertension. She had no history of Raynaud’s phenomenon, peripheral vascular disease, diabetes, or smoking.

Preoperative planning included discussion of observation, collagenase injection, and surgical excision. The patient complained of progressive discomfort due to the nodule, experiencing pain particularly when applying pressure on the hand against a flat surface or when gripping and holding objects. Given the increasing symptoms and functional limitation affecting daily activities, the patient elected to proceed with surgical excision under WALANT. Written informed consent was obtained after comprehensive counseling regarding the nature of the procedure, potential risks, benefits, and alternative treatment options.

On the day of surgery, 9 mL of 1% lidocaine with epinephrine (1:100,000) was infiltrated using a field block technique. One mL of sodium bicarbonate was added to buffer the solution. A dwell time of 40 minutes ensured optimal vasoconstriction and anesthesia 3 .

Surgery was performed via a Bruner incision, excising the Dupuytren’s nodule. Tenosynovitis was observed in both the deep and superficial flexor tendons, and a prophylactic A1 pulley release was performed to facilitate improved tendon gliding. Intraoperative perfusion appeared normal. The patient experienced an uneventful postoperative recovery and was subsequently discharged from the hospital.

Four hours postoperatively, the patient noticed increasing coldness and numbness of the digit, leading to urgent consultation in our emergency department. Examination revealed cyanosis distal to the proximal interphalangeal joint, absent capillary refill, and complete sensory loss, consistent with digital ischemia 6 , 9 .

Phentolamine was unavailable in our institution; 2.5 mL of urapidil (12.5 mg) was injected locally near the A1 pulley. Urapidil is accessible within our anesthesia service for use in the treatment of hypertensive emergencies. Within minutes, reperfusion was evident, with restoration of warmth, normal color, and sensation 7 , 8 . Systemic hemodynamics remained stable. The patient was monitored overnight; recovery was uneventful.

At two-week follow-up, the finger was well-perfused with normal motion and sensation. At six weeks, wound healing was complete, and the patient reported full satisfaction.

## Discussion

This case highlights several important considerations in the management of rare delayed-onset digital ischemia following WALANT. First, even in patients without evident vascular disease, advanced age and microvascular changes may increase susceptibility to prolonged vasospasm. Clinicians should be aware that ischemic complications can present hours after surgery, necessitating patient education regarding early warning signs, including persistent coldness, discoloration, or sensory loss 6 , 9 .

Epinephrine-induced vasoconstriction occurs through stimulation of α₁-adrenergic receptors in the vascular smooth muscle, resulting in decreased perfusion distal to the injection site. Normally, this effect is transient, and perfusion recovers spontaneously as the epinephrine is metabolized. However, in rare cases, vasospasm may persist or be exacerbated by patient-specific factors such as endothelial dysfunction, microvascular disease, or local tissue characteristics 2 , 3 .

Phentolamine is the traditionally recommended reversal agent due to its rapid α-adrenergic blockade and ability to restore digital perfusion effectively. Several case reports have demonstrated successful reversal of delayed ischemia even up to 14 hours post-injection 10 . In the absence of phentolamine, urapidil—a selective peripheral α₁-adrenergic antagonist with central sympatholytic effects–offers a useful alternative. Its advantages include potent peripheral vasodilation, minimal reflex tachycardia, and a favorable safety profile in elderly patients 7 , 8 . In our case, local infiltration of urapidil rapidly restored perfusion, suggesting that it may serve as an effective off-label rescue therapy in settings where phentolamine is unavailable.

From a practical standpoint, urapidil is generally more accessible in European hospitals than phentolamine. Marketed as Ebrantil or Urapidil Stragen, it is widely used for perioperative blood pressure control and hypertensive emergencies, as well as in patients requiring stable hemodynamic management without excessive cardiac workload or reflex tachycardia 11 . In Switzerland and other European countries, a pack of five 50 mg/10 mL ampoules typically costs between CHF 75–150 (€ 70–140), corresponding to approximately CHF 15–30 (€ 14–28) per ampoule, depending on supplier and regional availability 12 . In contrast, phentolamine (Regitine) is less frequently available and often requires special ordering, as it is no longer routinely marketed in several European countries. Where obtainable, its cost is typically higher–often CHF 40–60 (€ 38–56) per ampoule–and supply limitations further restrict its use in acute settings. Consequently, urapidil may represent not only a pharmacologically suitable but also a more practical and cost-effective alternative for the reversal of epinephrine-induced digital ischemia in the clinical environment.

From a clinical perspective, this case emphasizes several preventive and management strategies: preoperative screening for vascular risk factors, careful use of epinephrine in elderly or patients with vascular disease, standardized monitoring protocols post-WALANT procedures, patient education regarding ischemic symptoms, and ensuring immediate access to reversal agents. Additionally, the successful use of urapidil in this case warrants further investigation in prospective studies or animal models to determine optimal dosing, safety, and comparative efficacy with phentolamine.

Finally, while WALANT is generally safe and transformative in hand surgery, awareness of rare but potentially severe complications is critical. Expanding the repertoire of available reversal agents, including off-label options like urapidil, may enhance patient safety and provide clinicians with effective tools for managing digital ischemia.

## Conclusion

WALANT is a safe and transformative technique in hand surgery. Rare ischemic complications require vigilance and preparedness. Urapidil may serve as an effective alternative to phentolamine for epinephrine-induced digital ischemia. Early recognition and intervention are essential to ensure complete recovery 3 , 7 .

## Patient Consent Statement

Written informed consent was obtained from the patient for publication of this case report, including all clinical information and associated images. Confidentiality and anonymity were maintained.
