# Supplementary material for: Late-Onset Digital Ischaemia after WALANT: Successful Reversal with Off-Label Urapidil
Source: Handchir Mikrochir Plast Chir. 2025 Dec 11;58(3):273–6. doi: 10.1055/a-2739-3810 (PMC13253014; doi:10.1055/a-2739-3810)
Supplement: Related texts

# Research article: Verzögert auftretende digitale Ischämie nach WALANT: Erfolgreiche Behandlung mit Off-Label Einsatz von Urapidil

**Keywords:** WALANT, digitale Ischämie, Urapidil, Adrenalin, Handchirurgie

## Abstract

**Hintergrund:**

WALANT (Wide Awake Local Anesthesia No Tourniquet) hat die Handchirurgie durch die Kombination von Lidocain und Adrenalin ohne Sedierung und Blutleere wesentlich verändert. Obwohl niedrig dosiertes Adrenalin heute als sicher gilt, können in seltenen Fällen ischämische Komplikationen auftreten.

**Fallbericht:**

Wir berichten über eine 74-jährige Patientin, die nach der WALANT-gestützten Exzision eines Dupuytren-Knotens eine verspätet einsetzende digitale Ischämie entwickelte. Da Phentolamin – das Standardantidot – nicht verfügbar war, erfolgte die Infiltration mit Urapidil (12,5 mg), einem selektiven α₁-Blocker. Darunter kam es rasch zur Reperfusion und vollständigen Sensibilitätsrückkehr. Die Patientin erholte sich ohne bleibende Schäden.

**Schlussfolgerung:**

Dieser Fall verdeutlicht eine seltene, aber kritische Komplikation der WALANT-Technik. Urapidil könnte eine sichere und wirksame Alternative zu Phentolamin darstellen, wenn dieses nicht verfügbar ist.

## Einleitung

Die WALANT-Methode (Wide Awake Local Anesthesia No Tourniquet), erstmals durch Lalonde beschrieben 1 , hat die moderne Handchirurgie grundlegend verändert. Durch die Kombination von Lidocain mit niedrig dosiertem Adrenalin (typischerweise 1:100.000) kann sowohl eine wirksame Anästhesie wie auch Hämostase bewirkt werden, ohne Notwendigkeit einer Sedierung oder eines Tourniquets. Die Technik ermöglicht es, dass Patienten wach und führbar bleiben und erlaubt so eine intraoperative Beurteilung der aktiven Beweglichkeit, was insbesondere bei Sehnen- und Nervenoperationen nützlich ist. Daneben wird WALANT weltweit zunehmend eingesetzt, da es sich als kosteneffektiv erweist, die perioperative Morbidität reduziert und Eingriffe in kleineren Eingriffsräumen außerhalb des traditionellen Operationssaals ermöglicht 2 3 .

Der Einsatz von Adrenalin hat bei der WALANT-Anästhesie eine doppelte Funktion. Seine vasokonstriktive Wirkung reduziert erheblich die intraoperative Blutung, sorgt für ein klares Operationsfeld und verkürzt folglich die Operationszeit. Zudem verlangsamt Adrenalin die systemische Resorption von Lidocain und verlängert somit dessen anästhetische Wirkungsdauer und erhöht den Patientenkomfort während des Eingriffs. Die Dauer der Anästhesie beträgt üblicherweise 4–6 Stunden, abhängig von lokaler Gewebeeigenschaft und Injektionstechnik. Die maximale vasokonstriktive Wirkung tritt in der Regel 20–30 Minuten nach der Infiltration ein, weshalb vor dem Hautschnitt eine ausreichende Wartezeit für eine optimale Hämostase entscheidend ist 3 .

Initial bestanden Bedenken hinsichtlich des Risikos, durch die Injektion von Adrenalin digitale Nekrosen an Fingern oder Zehen zu verursachen. Diese entstanden häufig aufgrund von einzelnen Fallberichten, wobei es hier zum Einsatz von hochkonzentrierter Lösung oder nicht gepufferten Präparaten kam 4 5 . Zahlreiche Studien aus den letzten zwei Jahrzehnten haben jedoch die Sicherheit von korrekt gepuffertem, niedrig dosiertem Adrenalin in digitalen Blockaden bestätigt. Selbst bei älteren Patienten oder bei vaskulären Begleiterkrankungen zeigen sich nur sehr niedrige Raten ischämischer Komplikationen (<0,1%) 3 6 .

Trotz der nachgewiesenen hohen Anwendersicherheit erfordert der Einsatz von WALANT eine sorgfältige Patientenselektion. Kontraindikationen umfassen bekannte periphere Gefäßerkrankungen, Raynaud-Phänomen, Morbus Buerger, vorausgegangene digitale Replantationen, schwere Arteriosklerose, nicht eingestellte Hypertonie sowie systemische Erkrankungen, welche die Mikrozirkulation beeinträchtigen können 5 7 . Eine altersbedingte endotheliale Dysfunktion und verringerte mikrovaskuläre Reservefunktion können die Anfälligkeit für vasospastische Ereignisse trotz negativer Krankheitsanamnese erhöhen. Die präoperative Beurteilung sollte eine detaillierte vaskuläre Anamnese sowie die Erwägung alternativer Anästhesiemethoden bei Hochrisikopatienten beinhalten.

Phentolamin, ein nichtselektiver α-adrenerger Antagonist, wird weiterhin als Standard-Antidot für die Adrenalin-induzierte digitale Ischämie propagiert. Dies kann jedoch in manchen Institutionen nicht oder nur eingeschränkt verfügbar sein. Alternative pharmakologische Medikamente, darunter selektive α₁-Blocker wie Urapidil, sollten daher in Betracht gezogen werden. Ein grundlegendes Verständnis der zugrundeliegenden Ursachen für Vasospasmen, der pharmakologischen Wirkungen der Antidote und der Risikofaktoren für Ischämie ist entscheidend für die Patientensicherheit unter WALANT 7 8 .

## Fallbericht

Eine 74-jährige rechtsdominante Frau stellte sich mit einem Dupuytren-Knoten an der Basis ihres rechten Mittelfingers vor. Die Anamnese der Nebenerkrankungen ergab eine gut eingestellte arterielle Hypertonie. Es lagen keine Hinweise auf ein Raynaud-Phänomen, eine periphere Gefäßerkrankung, Diabetes oder Nikotinkonsum vor.

Die präoperative Planung beinhaltete die Diskussion über eine konservative abwartende Therapie, Kollagenase-Injektion und chirurgische Exzision. Die Patientin beklagte jedoch zunehmende, durch den Knoten bedingte Beschwerden in Form von Schmerzen beim Aufstützen der Hand auf eine flache Oberfläche oder beim Greifen und Halten von Gegenständen. Aufgrund der zunehmenden Symptomatik und insbesondere der funktionellen Einschränkung im Alltag entschied sich die Patientin für eine chirurgische Exzision unter WALANT. Nach umfassender Aufklärung über die Operationstechnik, mögliche Vorteile und Risiken sowie alternative Behandlungsoptionen wurde eine schriftliche Einwilligung eingeholt.

Am Operationstag wurden in Feldblocktechnik 9 ml 1%iges Lidocain mit Adrenalin (1:100.000) infiltriert. Ein Milliliter Natriumbicarbonat wurde als Pufferlösung hinzugefügt. Eine Wirkzeit von 40 Minuten gewährleistete eine optimale Vasokonstriktion und Anästhesie 3 .

Die Operation erfolgte über einen Bruner-Zugang unter Exzision des Dupuytren-Knotens. Es zeigte sich nebenbefundlich eine Tenosynovitis sowohl der tiefen wie auch der oberflächlichen Beugesehnen. Somit wurde zur Verbesserung der Sehnengleitfähigkeit intraoperativ eine prophylaktische A1-Ringbandspaltung durchgeführt. Die intraoperative Durchblutung erschien normal. Die postoperative Überwachung verlief komplikationslos, und die Patientin konnte anschließend in das ambulante Umfeld entlassen werden.

Vier Stunden postoperativ bemerkte die Patientin eine plötzlich zunehmende Kälte und Taubheit des operierten Fingers. Nach Rücksprache mit dem Dienstteam erfolgte eine sofortige Vorstellung in unserer Notaufnahme. Die Untersuchung zeigte eine Zyanose distal des proximalen Interphalangealgelenks, eine fehlende Rekapillarisierung des betroffenen Fingers und einen kompletten Sensibilitätsverlust – Befunde, die mit einer digitalen Ischämie vereinbar sind 6 9 .

Phentolamin war in unserem Krankenhaus nicht verfügbar; daher wurde entschieden, alternativ 2,5 ml Urapidil (12,5 mg) lokal in der Nähe des A1-Ringbands zu injizieren. Urapidil ist in unserer Anästhesieabteilung zur Behandlung hypertensiver Notfälle verfügbar. Innerhalb von Minuten zeigte sich eine Reperfusion mit Wiederherstellung der Wärme, normalem Kolorit und Sensibilität 7 8 . Die Patientin war stets hämodynamisch stabil und konnte nach stationärer Überwachung am Folgetag entlassen werden.

Bei einer Kontrolluntersuchung nach zwei Wochen zeigte der Finger eine regelrechte Perfusion mit normaler Beweglichkeit und Sensibilität. Nach sechs Wochen war die Wundheilung abgeschlossen, und die Patientin war mit dem Rehabilitationszustand zufrieden.

## Diskussion

Dieser Fall zeigt mehrere wichtige Aspekte in der Behandlung der sehr selten beschriebenen, verzögert auftretenden digitalen Ischämie nach WALANT auf. Erstens können selbst bei Patienten ohne relevante Gefäßerkrankung ein fortgeschrittenes Alter und eine mikrovaskuläre Veränderung das Risiko für einen verlängerten Vasospasmus erhöhen. Im klinischen Alltag sollte man sich darüber bewusst sein, dass ischämische Komplikationen auch mehrere Stunden nach der Intervention auftreten können. Deshalb ist eine gute Aufklärung der Patienten über Frühsymptome wie anhaltende Kälte, Verfärbung oder Sensibilitätsverlust entscheidend 6 9 .

Die Adrenalin-induzierte Vasokonstriktion erfolgt über die Stimulation von α₁-adrenergen Rezeptoren in der Gefäßmuskulatur, welche zu einer verminderten Perfusion distal der Injektionsstelle führt. Normalerweise ist dieser Effekt nur vorübergehend vorhanden und es kommt nach Metabolisierung des Adrenalins zu einer spontanen Erholung der Durchblutung. In seltenen Fällen kann es jedoch zu einem persistierenden Vasospasmus kommen. Unter Umständen wird dies durch patientenspezifische Faktoren, wie endotheliale Dysfunktion, mikrovaskuläre Erkrankung oder lokale Gewebeeigenschaften, verstärkt 2 3 .

Phentolamin wird klassischerweise aufgrund seiner schnellen α-Blockade und Fähigkeit, die digitale Perfusion effektiv wiederherzustellen, als Antidot empfohlen. Mehrere Fallberichte zeigen die erfolgreiche Reversibilität bis zu 14 Stunden nach erfolgter Injektion 10 . Falls Phentolamin nicht vorhanden ist, bietet Urapidil – ein selektiver peripherer α₁-Blocker mit zentral sympatholytischen Effekten – eine sinnvolle Alternative. Seine Wirkung äußert sich in einer ausgeprägten peripheren Vasodilatation, einer minimalen reflektorischen Tachykardie und einem günstigen Sicherheitsprofil bei älteren Patienten 7 8 . In unserem Fall führte die lokale Infiltration von Uradipil rasch zu einer Wiederherstellung der Perfusion, was darauf hindeutet, dass es als wirksame Off-Label-Rescue-Therapie in Situationen, in denen Phentolamin nicht verfügbar ist, eingesetzt werden könnte.

Aus praktischer Sicht ist Urapidil in europäischen Krankenhäusern häufig leichter verfügbar als Phentolamin. Unter den Handelsnamen Ebrantil oder Urapidil Stragen wird es verbreitet zur perioperativen Blutdruckkontrolle und bei hypertensiven Notfällen eingesetzt sowie bei Patienten, die ein stabiles hämodynamisches Management ohne übermäßige kardiale Belastung oder Reflextachykardie benötigen 11 . In der Schweiz und anderen europäischen Ländern kostet eine Packung mit fünf Ampullen à 50 mg/10 ml typischerweise 75–150 CHF (70–140 EUR), entsprechend etwa 15–30 CHF (14–28 EUR) pro Ampulle, je nach Anbieter und Verfügbarkeit 12 .

Im Gegensatz dazu ist Phentolamin (Regitin) seltener verfügbar und erfordert häufig eine Sonderbestellung, da es in mehreren europäischen Ländern nicht mehr routinemäßig vertrieben wird. Dort, wo es erhältlich ist, liegen die Kosten typischerweise höher – oft 40–60 CHF (ca. 38–56 EUR) pro Ampulle – und Lieferengpässe schränken seinen Einsatz in akuten Situationen zusätzlich ein. Folglich stellt Urapidil nicht nur eine pharmakologisch geeignete, sondern auch eine praktischere und kosteneffizientere Alternative zur Aufhebung einer durch Adrenalin induzierten Digitalischämie in der klinischen Praxis dar.

Aus klinischer Perspektive hebt dieser Fall mehrere präventive Maßnahmen und Managementstrategien hervor: präoperative Erfassung vaskulärer Risikofaktoren, sorgfältiger Einsatz von Adrenalin bei älteren Patienten oder solchen mit Gefäßerkrankungen, standardisierte Überwachungsprotokolle nach WALANT-Eingriffen, Aufklärung der Patienten hinsichtlich ischämischer Symptome sowie Sicherstellung des sofortigen Zugangs zu reversierenden Substanzen. Darüber hinaus rechtfertigt der erfolgreiche Einsatz von Urapidil in diesem Fall weitere Untersuchungen in prospektiven Studien oder Tiermodellen, um die optimale Dosierung, Sicherheit und vergleichende Wirksamkeit mit Phentolamin zu ermitteln.

Obwohl WALANT grundsätzlich sicher und wegweisend in der Handchirurgie ist, bleibt die Kenntnis seltener, potenziell schwerwiegender Komplikationen entscheidend. Die Verfügbarkeit zusätzlicher Antidotstrategien, inklusive Off-Label-Präparate wie Urapidil, kann die Patientensicherheit erhöhen und Ärzte mit effektiven Mitteln zur Behandlung einer Digitalischämie ausstatten.

## Schlussfolgerung

WALANT ist eine sichere und wegweisende Anästhesieform in der Handchirurgie. Seltene ischämische Komplikationen erfordern Wachsamkeit und eine gute Vorbereitung. Urapidil kann als wirksame Alternative zu Phentolamin bei Adrenalin-induzierter digitaler Ischämie dienen. Früherkennung und Intervention sind entscheidend für eine vollständige Genesung 3 7 .

## Einverständniserklärung der Patientin

Eine schriftliche Einwilligung der Patientin zur Veröffentlichung dieses Fallberichts einschließlich aller klinischen Informationen wurde eingeholt. Vertraulichkeit und Anonymität wurden gewährleistet.
